# Structure-Aided Identification of an Inhibitor Targets Mps1 for the Management of Plant-Pathogenic Fungi

**DOI:** 10.1128/mbio.02883-22

**Published:** 2023-02-13

**Authors:** Zhiwei Kong, Xin Zhang, Feng Zhou, Liu Tang, Yitong Chen, Saijie Li, Xiaokang Zhang, Letian Kuai, Wenji Su, Weiren Cui, Jiaxi Cai, Yuli Wang, Jun Yang, You-Liang Peng, Dongli Wang, Junfeng Liu

**Affiliations:** a Ministry of Agriculture Key Laboratory for Crop Pest Monitoring and Green Control, College of Plant Protection, China Agricultural University, Beijing, China; b WuXi AppTec (Shanghai) Co., Ltd., Shanghai, China; c Joint International Research Laboratory of Crop Molecular Breeding, China Agricultural University, Beijing, China; Columbia University

**Keywords:** crystal structure, *Magnaporthe oryzae*, MAP kinase, inhibitor, fungicide

## Abstract

Blast disease caused by Magnaporthe oryzae threatens rice production worldwide, and chemical control is one of the main methods of its management. The high mutation rate of the M. oryzae genome results in drug resistance, which calls for novel fungicide targets. Fungal proteins that function during the infection process might be potential candidates, and Mps1 (M. oryzae mitogen-activated protein kinase 1) is such a protein that plays a critical role in appressorium penetration of the plant cell wall. Here, we report the structure-aided identification of a small-molecule inhibitor of Mps1. High-throughput screening was performed with Mps1 against a DNA-encoded compound library, and one compound, named A378-0, with the best performance was selected for further verification. A378-0 exhibits a higher binding affinity than the kinase cosubstrate ATP and can inhibit the enzyme activity of Mps1. Cocrystallization of A378-0 with Mps1 revealed that A378-0 binds to the catalytic pocket of Mps1, while the three ring-type substructures of A378-0 constitute a triangle that squeezes into the pocket. *In planta* assays showed that A378-0 could inhibit both the appressorium penetration and invasive growth but not the appressorium development of M. oryzae, which is consistent with the biological function of Mps1. Furthermore, A378-0 exhibits binding and activity inhibition abilities against Mpk1, the Mps1 ortholog of the soilborne fungal pathogen Fusarium oxysporum. Collectively, these results show that Mps1 as well as its orthologs can be regarded as fungicide targets, and A378-0 might be used as a hit compound for the development of a broad-spectrum fungicide.

## INTRODUCTION

Magnaporthe oryzae (syn., Pyricularia oryzae) is the filamentous fungus that causes rice blast, one of the most devastating diseases of cultivated rice (1). M. oryzae was first reported by Cavara in 1891 (2). Besides rice, M. oryzae infects more than 50 plants of the grass family Poaceae, including wheat (Triticum aestivum), foxtail millet (Setaria italica), and grasses (Eleusine indica and Setaria viridis, etc.) (3, 4). It is believed that the rice-infecting lineage of M. oryzae emerged following a host shift from S. italica about 2,500 to 7,500 years ago (4). Due to their shorter generation times and higher mutation rates, M. oryzae strains have adapted to various rice species via the gain and loss of genes, rapid differentiation in pathogenicity-related genes, changing transposon elements, and sequence divergence, etc. (5–7). To date, no effective long-term solution has been found for combating rice blast, with chemical control still playing a vital role in guaranteeing rice production (2). However, with emerging drug resistance, identifying new fungicide targets is an urgent need for combating rice blast (8).

Understanding the life cycle of M. oryzae is important for determining promising fungicide targets. M. oryzae disperses via conidia with wind and dewdrop splash (9). After adhering to the rice leaf surface, the conidia differentiate into appressoria, dome-shaped infection cells that can accumulate pressure to penetrate the plant cell wall and develop into invasive hyphae (10). Therefore, the host penetration period of M. oryzae is critical for its successful invasion of a plant host, and M. oryzae proteins that function during this period may serve as promising targets for fungicide development (11).

Kinases catalyze the transfer of the γ-phosphate of ATP to serine, threonine, or tyrosine residues of a substrate protein, resulting in signal transduction in eukaryotic living cells (12). In the human genome, 518 kinases have been identified, which modify up to one-third of the proteome, and there are about 90 small-molecule kinase inhibitors targeting about 20 kinases that have been clinically approved for disease treatment (https://www.ppu.mrc.ac.uk/) (13). However, for fungicides, only seven small molecules have been reported, targeting the kinase HOG1 (high-osmolarity glycerol 1) or Daf1 (decay-accelerating factor 1) (www.frac.info/). Therefore, other kinases of fungal pathogens might be developed as novel fungicide targets.

M. oryzae contains three mitogen-activated protein (MAP) kinases, Mps1, Pmk1, and Osm1 (14, 15). Mps1 plays a role in appressorium penetration and invasive hyphal growth periods but does not influence the formation of appressoria (16, 17). Specifically, the deletion of *Mps1* results in the complete loss of pathogenicity, as the appressoria are unable to penetrate plant cell surfaces, albeit the formation of appressoria is not influenced (17). Pmk1 functions during appressorium formation, appressorium penetration, and invasive hyphal growth periods and controls the cell-to-cell invasion of rice cells by fungi (10, 16, 18–20). Osm1 suppresses host innate immunity by participating in reactive oxygen species (ROS) scavenging during the invasion process (21). Therefore, all three of the MAP kinases of M. oryzae are important for pathogen virulence and might be considered novel fungicide targets to search for inhibitors for rice blast control. In this study, we chose Mps1 as a target for inhibitor screening and characterization and identified an inhibitor, named A378-0, with a novel chemical structure and verified biological activity, which may be considered a favorable starting molecule for further fungicide design and development.

## RESULTS

### Identification of an inhibitor of Mps1 by library screening.

**FIG 1:**
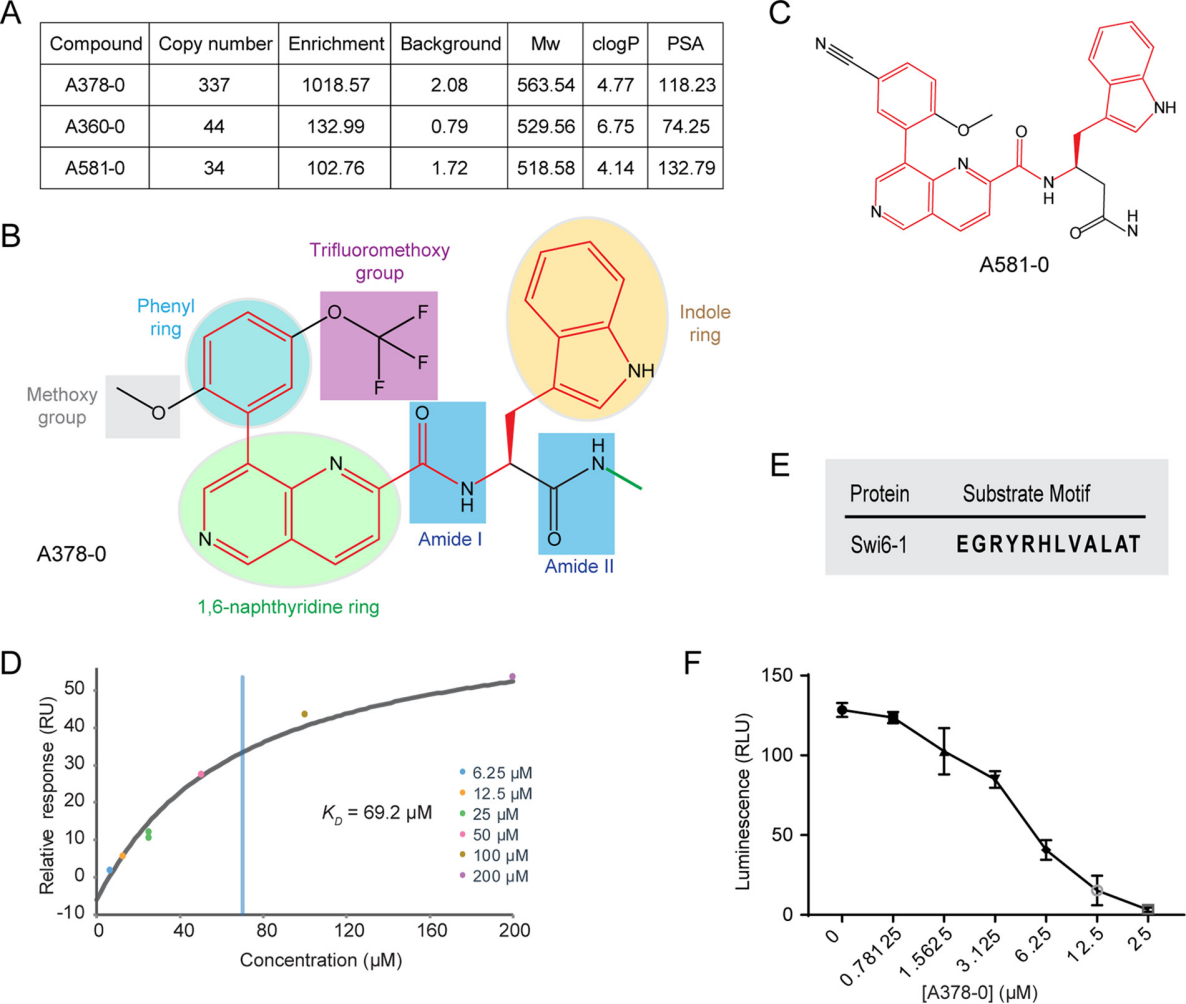
Identification of compound A378-0 as an inhibitor of Mps1. (A) The final compound list screened with Mps1. (B) Chemical structure of compound A378-0. A378-0 consists mainly of three ring structures and four other functional groups. The two amide groups are numbered I and II. Red highlights the scaffold of A378-0, and the green bond indicates the bond that is linked to DNA. (C) Chemical structure of compound A581-0, with red highlighting the same scaffold as the one for A378-0. (D) A378-0 binds Mps1 *in vitro*. The experiment was carried out with the SPR method. The blue vertical line indicates the *K_D_* value. The ATP analog AMP-PNP was used as a negative control (see Fig. S2 in the supplemental material). (E) The kinase substrate motif of Swi6 (named Swi6-1) used as the substrate of Mps1. (F) A378-0 inhibits the kinase activity of Mps1. The *y* axis indicates enzyme activity. Error bars indicate standard deviations from three replicates. RLU, relative luminescence units.

We screened for inhibitor compounds against Mps1 with a DNA-encoded compound library (DEL) named DELopen (WuXi AppTec) (see Fig. S1A and B in the supplemental material). This library contains about 4.4 billion compounds, all of which have been newly synthesized using split-and-pool combinatorial chemistry (22). Each compound is conjugated through an amide group with a specific DNA barcode (a fragment of DNA with a specific sequence), the sequencing of which can be used to identify its ligated compound (Fig. S1A). The screening procedure is shown in Fig. S1B. With a suggested cutoff of an enrichment value of >100, we obtained a list of 206 chemical compounds. However, by eliminating those with copy numbers of <10 or background values of >20, there were only three compounds left, A378-0, A360-0, and A581-0 (Fig. 1A). Of the three compounds, A378-0 has the best copy number and enrichment value; meanwhile, its molecular weight, calculated octanol-water partition coefficient (clogP), and polar surface area (PSA) are all acceptable according to the “rule of five” (Fig. 1A) (23). Besides, although bearing the same scaffold as A378-0, A581-0 has different substituent groups and exhibits lower copy numbers and enrichment values than A378-0 (Fig. 1A to C). Therefore, we chose only A378-0 for further analysis.

10.1128/mbio.02883-22.1 FIG S1 Screening of small-molecule compound inhibitors with a DNA-encoded compound library. (A) Schematic diagram of DNA-labeled compounds of the DNA-encoded compound library. Each compound is linked to a DNA barcode, the sequence of which is used to identify the compound. (B) Inhibitor screening procedure with a DNA-encoded compound library. Download FIG S1, TIF file, 0.3 MB. Copyright © 2023 Kong et al. 2023 Kong et al. https://creativecommons.org/licenses/by/4.0/ This content is distributed under the terms of the Creative Commons Attribution 4.0 International license.

10.1128/mbio.02883-22.2 FIG S2 SPR assay of the affinity between AMP-PNP and Mps1. (A) Comparison of the ATP and AMP-PNP chemical structures. The difference between them is highlighted in orange. (B) SPR sensograms. The results show that AMP-PNP at a concentration range between 15.625 μM and 500 μM does not interact with Mps1. Download FIG S2, TIF file, 0.4 MB. Copyright © 2023 Kong et al. 2023 Kong et al. https://creativecommons.org/licenses/by/4.0/ This content is distributed under the terms of the Creative Commons Attribution 4.0 International license.

Compound A378-0 features three ring-type structures, a phenyl ring, a 1,6-naphthyridine ring, and an indole ring, that constitute the backbone of the compound (Fig. 1B). In addition, there are other functional groups or substituents, including two amide groups (numbered I and II), a methoxy group, and a trifluoromethoxy group (Fig. 1B). Actually, amide group II serves as the amide bond that links A378-0 and DNA (Fig. 1B). This compound contains four oxygen atoms and five nitrogen atoms, and its clogP and aqueous solubility (logS) values are 4.242 and −7.436, respectively, suggesting its preferable solubility (Fig. 1B). We then synthesized A378-0 for further evaluation (Fig. S1B). Surface plasmon resonance (SPR) assays showed that the *in vitro* binding affinity (represented by the dissociation equilibrium constant [*K_D_*]) between A378-0 and Mps1 is 69.2 μM, confirming the interaction between them (Fig. 1D). Since ATP, the cosubstrate and phosphate donor source of kinase, is readily hydrolyzed in water solution, we used its nonhydrolyzable analog adenylyl imidodiphosphate (AMP-PNP) to evaluate the interaction between ATP and Mps1 by SPR (Fig. S2A). At a series of concentrations of between 15.625 μM and 500 μM AMP-PNP, no binding was detected, indicating that the *K_D_* between AMP-PNP and Mps1 is lower than 500 μM (Fig. S2B). It has been reported that the affinities between AMP-PNP and the kinases Itk and Src are 7.9 mM and 2.54 mM, respectively, as determined by fluorescence anisotropy, which fall into the estimated affinity range for AMP-PNP and Mps1 (24). Therefore, the affinity between Mps1 and A378-0 is much higher than that between Mps1 and AMP-PNP or ATP. Furthermore, we verified whether A378-0 could regulate the enzyme activity of Mps1. Swi6 functions downstream of Mps1 (25). With a synthesized peptide of Swi6 (Swi6-1) containing the signature kinase substrate motif as the substrate, we found that supplementation with A378-0 suppressed the kinase activity of Mps1 (Fig. 1E and F). Collectively, we obtained compound A378-0 that could bind Mps1 and inhibit its enzyme activity.

### Overall structure of the Mps1/A378-0 complex.

**FIG 2:**
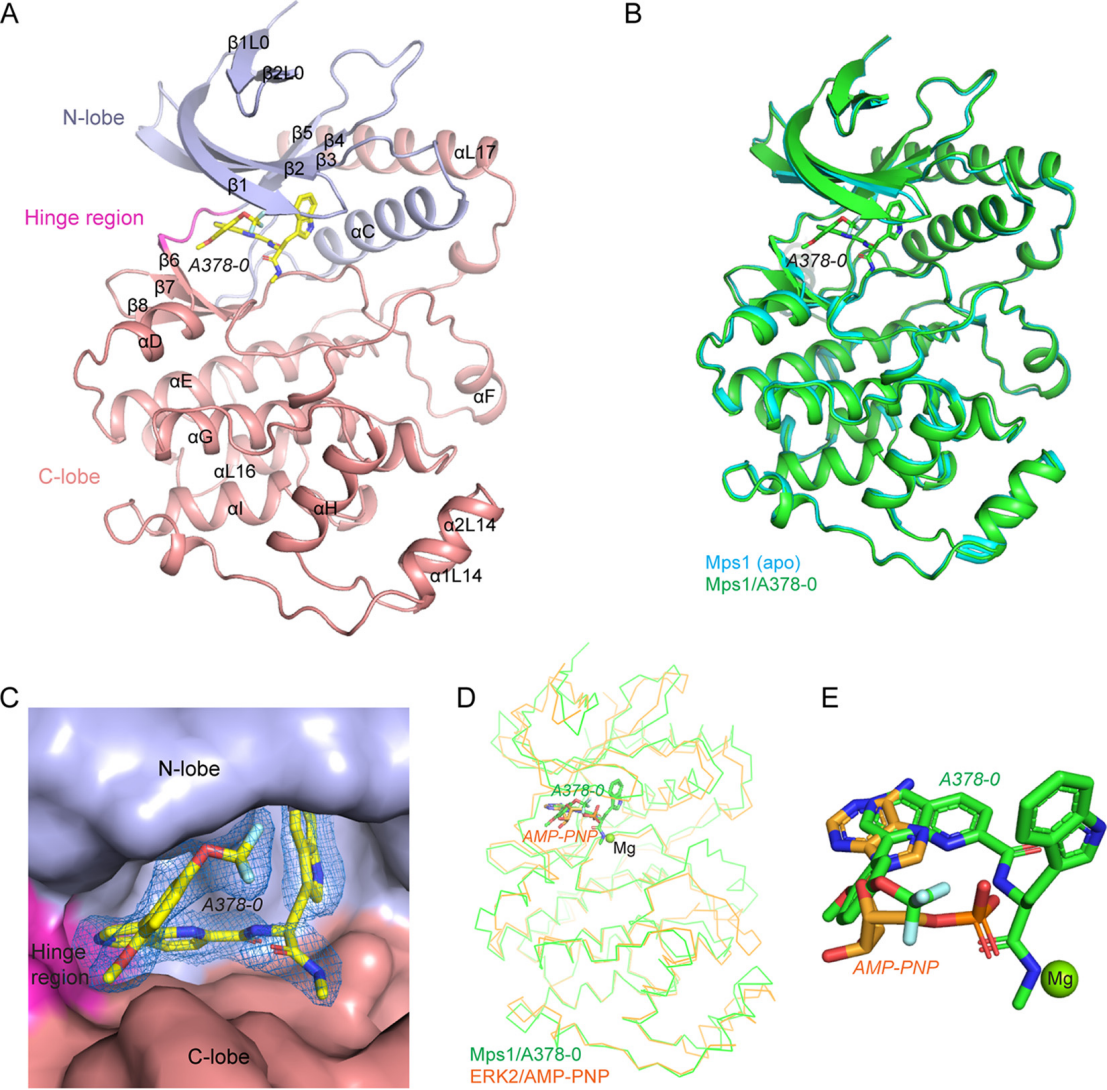
Crystal structure of the Mps1/A378-0 complex. (A) Overall structure of the Mps1/A378-0 complex. The N-lobe, hinge region, and C-lobe of Mps1 are shown in light blue, pink, and salmon, respectively. A378-0 is shown as sticks and colored by atom types, with yellow for carbon, blue for nitrogen, red for oxygen, and cyan for fluorine. (B) Structural superimposition of the Mps1/A378-0 complex with the Mps1 apo structure (PDB accession number 5Z33). Proteins are shown as cartoons and are colored differently. (C) Close-up view of compound A378-0 in the catalytic pocket of Mps1. The 2mF_o_-DF_c_ electron density map (contoured at 1.0 σ within 1.5 Å) of compound A378-0 is shown as a blue mesh. The surface of Mps1 is shown. The N-lobe, hinge region, and C-lobe are colored as described above for panel A. (D) Structural superimposition of the Mps1/A378-0 complex with the human ERK2/AMP-PNP complex (PDB accession number 4S32). Mps1 and ERK2 are shown as ribbons. A378-0 and AMP-PNP are shown as sticks. (E) Close-up view of A378-0 and AMP-PNP from panel D with a counterclockwise rotation of 45° around the *x* axis. The β- and γ-phosphates of AMP-PNP were not built into the ERK2/AMP-PNP complex structure. In panels D and E, the magnesium ion in the ERK2/AMP-PNP complex that is essential for enzyme activity is shown as a lime sphere. In panel E, amide group II of A378-0 occupies the corresponding position of the magnesium ion.

We then attempted to cocrystallize A378-0 with Mps1 to understand their interaction. Crystals were obtained under the crystallizing conditions of the apo-Mps1 crystal (Protein Data Bank [PDB] accession number 5Z33), with subtle modifications. The structure was determined at a 2.15-Å resolution. Each asymmetric unit contains one protein molecule. Residues Q5 to T360 and Q394 to D412 of Mps1 were built into the model, while others were not due to the invisibility of the corresponding electron density (Fig. 2A and Table 1). Structural superimposition with apo-Mps1 shows a root mean square deviation (RMSD) of 0.311 Å, indicating that only subtle conformational changes occurred in the newly determined structure (Fig. 2B).

**TABLE 1:** Diffraction data processing and structural refinement statistics of the Mps1/A378-0 complex structure

Parameter	Value^*a*^
Data collection and processing statistics	
Beamline	SSRF BL19U1
Wavelength (Å)	0.9792
Space group	P3_2_21
Cell dimensions	
*a*, *b*, *c* (Å)	75.04, 75.04, 159.06
α, β, γ (°)	90, 90, 120
Resolution (Å)	50.00–2.15
No. of unique reflections	28,949 (2,830)
*R*_merge_	0.146 (0.937)
CC_1/2_	0.984 (0.915)
*I*/σ(*I*)	30 (3.5)
Completeness (%)	99.87 (100.00)
Redundancy	19.4 (20.4)
Refinement statistics	
Resolution (Å)	30.08–2.15 (2.23–2.15)
*R*_work_	0.181
*R*_free_	0.205
No. of nonhydrogen atoms	3,214
Protein	3,023
Ligand	65
Water	150
RMSD	
Bond lengths (Å)	0.013
Bond angles (°)	1.02
Ramachandran plot (%)	
Favored regions	97.30
Allowed regions	2.70
Outliers	0.00
*B*-factors (Å^2^)	
Avg	48.12
Protein	48.27
Ligand	36.55
Water	48.43

a Values for the outer shell are given in parentheses.

As a typical kinase, the overall structure of Mps1 can be divided into two domains, a smaller N-terminal lobe (N-lobe, residues Q5 to E104) and a larger C-terminal lobe (C-lobe, residues C109 to D412), with a hinge region (residues E105 to E108) connecting them (Fig. 2A). The N-lobe of Mps1 contains seven β-strands (β1L0, β2L0, and β1 to β5, numbered according to protein kinase A (PKA) here and below) and one α-helix (the αC-helix) (26). The C-lobe consists of 3 β-strands (β6 to β8) and 10 α-helices (αD to αI, α1L14, α2L14, αL16, and αL17) (Fig. 2A). The deep cleft between the N-lobe and the C-lobe serves as the catalytic pocket for binding the cosubstrate ATP and substrates (Fig. 2A).

Extra electron density was observed in the catalytic pocket, readily fitted with compound A378-0 (Fig. 2A and C). A378-0 exhibits a compact conformation for squeezing into the pocket: the indole ring is oriented perpendicular to the central 1,6-naphthyridine ring, the benzene ring tilts about 45° from the 1,6-naphthyridine ring, and thus, the planes of the three rings approximate an equilateral right triangle (Fig. 2C). During the screening of the DNA-encoded compound library, the DNA barcode was conjugated to amide group II of A378-0; in the Mps1/A378-0 complex structure, amide group II resides at the mouth of the binding pocket of Mps1, and thus, the link of A378-0 with DNA would not block the binding of A378-0 with Mps1 (Fig. 1B and Fig. 2C). Compared with the human extracellular signal-regulated kinase 2 (ERK2)/AMP-PNP complex, we can see that the 1,6-naphthyridine ring of A378-0 is in the same plane as the AMP-PNP adenine, and the benzene ring of A378-0 resides in almost the same space as the AMP-PNP ribose, while the indole ring and the two amide groups of A378-0 stretch upward and occupy much more space (Fig. 2D and E). Besides, there is a magnesium ion in the ERK2/AMP-PNP complex structure, whereas amide group II of A378-0 occupies the corresponding position of the magnesium ion (Fig. 2D and E).

### Interactions between A378-0 and Mps1.

**FIG 3:**
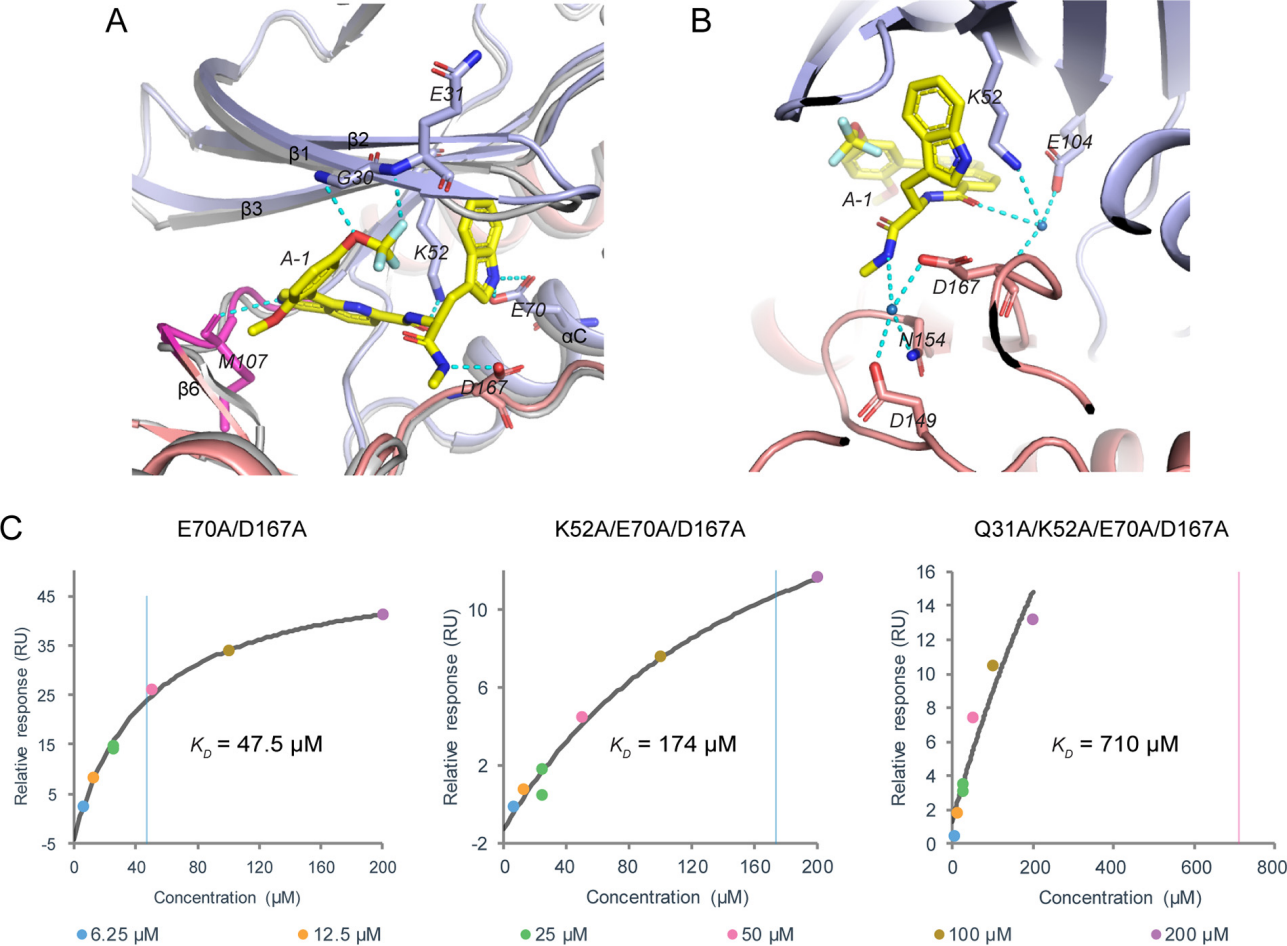
Hydrogen bond interactions between A378-0 and Mps1. (A) Direct intermolecular hydrogen bonds between A378-0 and Mps1. The superimposed apo-Mps1 structure (PDB accession number 5Z33) is shown in gray. (B) Indirect intermolecular hydrogen bonds between A378-0 and Mps1. Water molecules are shown as marine spheres. In panels A and B, Mps1 and A378-0 are shown and colored as described in the legend of Fig. 2A; residues of Mps1 participating in hydrogen bond interactions are labeled, with the side chain or main chain (if involved) shown as sticks; hydrogen bonds are shown as cyan dashed lines. (C) SPR assay results for the interactions between A378-0 and Mps1 double, triple, and quadruple mutants. The series of concentrations of A378-0 used are shown at the bottom of the charts.

Both hydrogen-bonding interactions and hydrophobic interactions play roles in the interaction between A378-0 and Mps1. There are seven intermolecular hydrogen bonds between A378-0 and Mps1 (Fig. 3A). First, the oxygen atom and one fluorine atom of the A378-0 trifluoromethoxy group form two hydrogen bonds with the main-chain nitrogen atoms of residues G30 and Q31, respectively (Fig. 3A). Residues G30 and Q31 belong to the glycine-rich loop (G-loop) between strands β1 and β2 of Mps1, and the formation of the two hydrogen bonds results in an extension of the β1-strand compared with apo-Mps1 (Fig. 3A). Second, one nitrogen atom of the 1,6-naphthyridine ring interacts with the main-chain oxygen of residue M107 of the hinge region of Mps1, which leads to a conformational change of the hinge and the emergence of the β6-strand compared with apo-Mps1 (Fig. 3A). Third, amide group I of A378-0 uses its oxygen atom to interact with the side chain nitrogen of residue K52 of the β3-strand of Mps1, while amide group II of A378-0 uses its nitrogen atom to interact with the side chain oxygen of residue D167 of the DFG motif of Mps1, rendering stabilization of residue D167 to point inward, i.e., Mps1 in the “DFG-aspartate-in” state (the side chain of the aspartate of the DFG motif points to the cleft) (Fig. 3A) (12). Finally, the last two hydrogen bonds occur between the nitrogen atom of the indole ring of A378-0 and the two oxygen atoms of the side chain carboxyl group of residue E70 of Mps1, which stabilizes the αC-helix in the “αC-helix-in” conformation (the side chain of the glutamate of the αC-helix points to the cleft) (Fig. 3A) (12).

In addition to these directly formed hydrogen bonds between A378-0 and Mps1, there are water-mediated intermolecular hydrogen-bonding interactions that involve two water molecules. One water molecule mediates the interaction between the nitrogen atom of amide group II of A378-0 and three side chain oxygen or nitrogen atoms: oxygen atoms of residues D149 and D167 and the nitrogen atom of residue N154 (Fig. 3B). The other water molecule bridges interactions from the oxygen atom of amide group I of A378-0 to the side chain nitrogen atom of residue K52, the side chain oxygen atom of residue E104, and the main-chain nitrogen atom of residue D167 (Fig. 3B). Both of the two water molecules interact with four nitrogen or oxygen atoms, forming two pyramid-like architectures that lead to improved three-dimensional stability (Fig. 3B).

Besides hydrogen bonds, hydrophobic interactions also function in stabilizing A378-0 in the cleft between the N- and C-lobes of Mps1 (Fig. S3A and B). These interactions happen between carbon or fluorine atoms of A378-0 and carbon atoms of Mps1 residues of the G-loop, the β1- to β3-strands, and the newly emerged β7-strand. For example, the trifluoromethoxy group of A378-0 uses its two fluorine atoms and one carbon atom to interact with residue V37; the phenyl ring of A378-0 interacts with residues L29 and V37; the 1,6-naphthyridine ring of A378-0 interacts with residues L29, A50, and L156; and the indole ring of A378-0 interacts with residues Y34, V37, and V54.

10.1128/mbio.02883-22.3 FIG S3 Hydrophobic interactions between A378-0 and Mps1. (A) Interactions calculated with LIGPLOT, with hydrogen bond information removed. Nonpolar or aromatic residues are shown in red and are regarded as residues for forming hydrophobic interactions with A378-0. (B) Three-dimensional view. A378-0 and Mps1 are shown and colored as described in the legend of Fig. 2A. Critical residues of Mps1 involved in hydrophobic interactions are shown as lines and labeled. Download FIG S3, TIF file, 1.4 MB. Copyright © 2023 Kong et al. 2023 Kong et al. https://creativecommons.org/licenses/by/4.0/ This content is distributed under the terms of the Creative Commons Attribution 4.0 International license.

From the interaction analysis described above, we can see the two substructional groups of the phenyl ring of A378-0 participate in the formation of both hydrogen bonds and hydrophobic interactions with Mps1, which can explain why compound A581-0 exhibited a lower copy number and a lower enrichment value, i.e., a lower binding affinity, than A378-0 during library screening (Fig. 1A to C).

We then carried out mutational studies of Mps1 to evaluate representative critical residues that are involved in interactions between A378-0 and Mps1 (Fig. 3C and Fig. S4). Residues Q31, K52, E70, and D167, which are involved in hydrogen bonds, and V37 and L156, which participate in hydrophobic interactions, were chosen and mutated to alanine. The affinity between A378-0 and the mutant proteins was determined by SPR. Probably due to the binding affinity between A378-0 and Mps1 being rather high and because many residues contribute to their interactions simultaneously, no obvious differences in affinity were observed in these single mutants, except for the two negatively charged residues E70 and D167 (Fig. S4). Therefore, we then carried out multiple mutations, including double mutation, triple mutation, and quadruple mutation, of the four residues contributing to hydrogen bonds (Fig. 3C). As expected, the SPR assay showed a substantial decrease in the binding affinity, especially for the quadruple mutant, the *K_D_* value of which exceeded the measurement limit of the Biacore 8k+ instrument. Therefore, it can be concluded that the four residues Q31, K52, E70, and D167 of Mps1 are important for its binding to A378-0, which also verified the accuracy of the Mps1/A378-0 structural model.

10.1128/mbio.02883-22.4 FIG S4 SPR assay results for the interactions between A378-0 and Mps1 single mutants. The series of concentrations of A378-0 used are shown at the bottom of the charts. Download FIG S4, TIF file, 0.8 MB. Copyright © 2023 Kong et al. 2023 Kong et al. https://creativecommons.org/licenses/by/4.0/ This content is distributed under the terms of the Creative Commons Attribution 4.0 International license.

### Bioactivity evaluation of A378-0.

**FIG 4:**
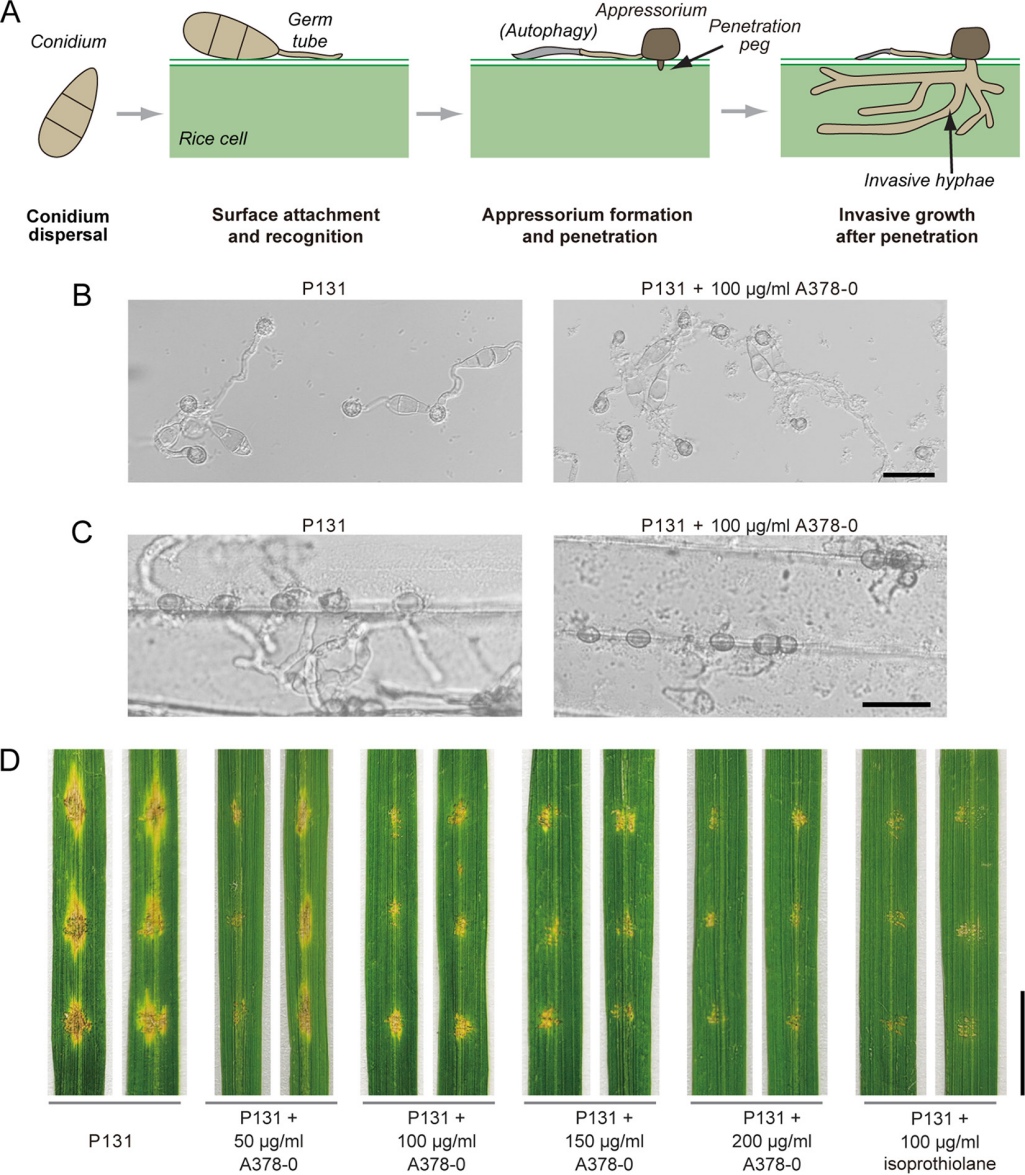
Compound A378-0 inhibits M. oryzae infection of hosts. (A) Schematic diagram of the process of M. oryzae infecting host cells. The image was made based on results reported previously by Jiang et al., with modifications (16). (B) A378-0 could not inhibit the appressorium development of M. oryzae. The assay was carried out on hydrophobic borosilicate glass coverslips. (C) A378-0 inhibits the development of M. oryzae conidia into penetration pegs and invasive hyphae. The assay was carried out with rice leaves. Bars = 25 μm (B and C). (D) Compound A378-0 inhibits M. oryzae virulence on rice leaves. Isoprothiolane was used as a positive control. Bar = 1 cm.

We then verified whether A378-0 possesses bioactivity in suppressing M. oryzae virulence. Infection of plants by M. oryzae begins with appressorium development; i.e., M. oryzae conidia recognize and adhere to rice hydrophobic surfaces, germinate, and develop into dome-shaped infection cells, appressoria. Later, in the presence of gradually accumulating turgor pressure, a penetration peg is formed to penetrate the host cell wall, and the cells differentiate into primary hyphae and then invasive hyphae, which grow from cell to cell and result in visible lesions (Fig. 4A) (9). Accordingly, we conducted experiments to evaluate the performance of A378-0 on the infection process of M. oryzae (Fig. 4B to D and Fig. S5A).

10.1128/mbio.02883-22.5 FIG S5 A378-0 inhibits M. oryzae virulence and other processes. (A) A378-0 inhibits M. oryzae virulence on barley leaves. Isoprothiolane was used as a positive control. Bar = 1 cm. (B) Conidia per petri dish produced by M. oryzae on OTA plates in the presence or absence of A378-0. Error bars indicate standard deviations from three independent experiments. (C) Colonies formed by M. oryzae on OTA plates at 5 days postinoculation in the presence or absence of A378-0. (D) Sensitivity of M. oryzae to cell wall-digesting enzymes in the presence or absence of A378-0. Photos were taken after 1 h of treatment with 7 mg mL^−1^ lysing enzymes from Trichoderma harzianum. Bar = 20 μm. Download FIG S5, TIF file, 10.6 MB. Copyright © 2023 Kong et al. 2023 Kong et al. https://creativecommons.org/licenses/by/4.0/ This content is distributed under the terms of the Creative Commons Attribution 4.0 International license.

With a hydrophobic glass surface mimicking the hydrophobic surface of plants, we found that A378-0 could not inhibit the germination and appressorium formation of M. oryzae conidia (Fig. 4B). On the other hand, after inoculation onto rice leaf sheaths for 36 h, the conidia of M. oryzae did not form a penetration peg in the presence of 100 μg mL^−1^ of A378-0, whereas conidia of the control group readily developed into invasive hyphae in rice cells, indicating that A378-0 could inhibit the appressorium penetration of M. oryzae (Fig. 4C). Actually, the two phenomena are in accordance with research showing that Mps1 does not function in appressorium development but functions in the progression of appressorium penetration (17).

We then evaluated whether A378-0 could inhibit the virulence of an M. oryzae strain *in planta*, with isoprothiolane [diisopropyl 2-(1,3-dithiolan-2-ylidene)malonate] as a control. Isoprothiolane is a fungicide that suppresses phospholipid biosynthesis and can inhibit the appressorium penetration of M. oryzae at a concentration of 20 μg mL^−1^ (27). In inoculation assays with conidia on rice leaves, we found that beginning with a concentration of 50 μg mL^−1^, inhibition of M. oryzae virulence by A378-0 can be observed: as the concentration of A378-0 increased, M. oryzae conidia gradually lost pathogenicity, and when the A378-0 concentration reached 150 μg mL^−1^, M. oryzae conidia were completely nonpathogenic (Fig. 4D). Besides its natural host, rice, the M. oryzae strain that we used also infects barley. Due to leaf surface properties, infection of rice leaves by M. oryzae conidia requires subtle friction before inoculation, whereas barley leaves do not need such a pretreatment, which preserves the intact cuticular layer. Similarly, A378-0 exhibits inhibition of M. oryzae virulence on barley leaves (Fig. S5A).

Mps1 is also important for other processes of M. oryzae, such as conidiation and cell wall integrity (17). We then evaluated whether A378-0 influences these processes. In the presence of 100 μg mL^−1^ A378-0, the conidiation of M. oryzae was reduced to about 40% (Fig. S5B). With supplementation with 50 μg mL^−1^ or 100 μg mL^−1^ A378-0, progressive autolysis was observed on the culture dish (Fig. S5C). In addition, after being cultured with 100 μg mL^−1^ A378-0, M. oryzae cells were readily reduced to spheroplasts after treatment with a lysing enzyme (Fig. S5D). Collectively, these assays showed that A378-0 can inhibit the virulence of M. oryzae by specifically targeting Mps1.

### A378-0 may serve as a hit for broad-spectrum fungicide development.

**FIG 5:**
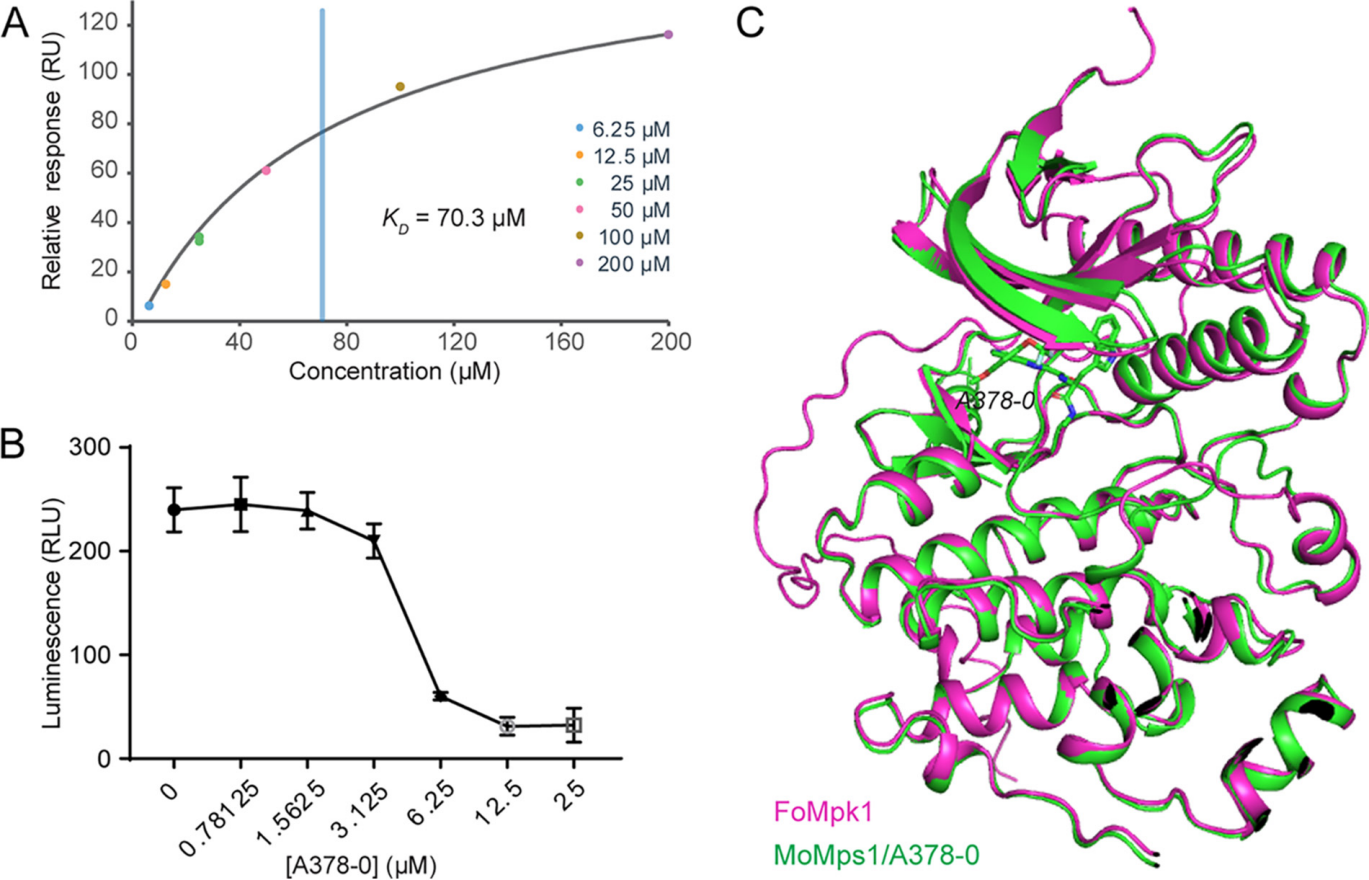
A378-0 is a potent inhibitor of Mpk1. (A) A378-0 binds Mpk1 *in vitro*. The experiment was carried out with the SPR method. The blue vertical line indicates the *K_D_* value. (B) A378-0 inhibits the kinase activity of Mpk1. Error bars indicate standard deviations from three replicates. (C) Structural superimposition of the Mpk1 model (AlphaFold2 model downloaded from the UniProt Knowledge Base) with the Mps1/A378-0 complex.

We then wondered whether A378-0 can inhibit the activity of Mps1 orthologs of other plant-pathogenic fungi. The sequence identity between Mps1 and its orthologs in such fungi, including Botrytis cinerea, Colletotrichum gloeosporioides, Fusarium graminearum, Fusarium oxysporum, and Sclerotinia sclerotiorum, ranges from 87.8% to 91.0% (Fig. S6). F. oxysporum is a soilborne pathogen that plays a vital role in causing root rot of plants such as Panax notoginseng, an important traditional Chinese medicinal material (28). The Mps1 ortholog of F. oxysporum is Mpk1, which is involved in the regulation of the development, stress response, and virulence of F. oxysporum in both animals and plants (29). We then expressed recombinant Mpk1 and examined the performance of A378-0 on it. SPR assays showed a *K_D_* of 70.3 μM between A378-0 and Mpk1, which is comparable to that between A378-0 and Mps1 (Fig. 5A). Enzymatic assays suggested that A378-0 can inhibit the kinase activity of Mpk1 (Fig. 5B). We then compared the model of Mpk1 with that of our Mps1/A378-0 complex (Fig. 5C). The RMSD between Mpk1 and Mps1 was 0.525 Å, supporting their structural similarity (Fig. 5C). Therefore, besides M. oryzae, A378-0 may also serve as a hit for fungicide development for the management of other fungal plant pathogens such as F. oxysporum.

10.1128/mbio.02883-22.6 FIG S6 Sequence alignment of Mps1 with its orthologs from fungal plant pathogens and the other MAP kinases of M. oryzae. The following sequences were used: Bmp3 from Botrytis cinerea (GenBank accession number ABJ51957.1), Slt2 from Colletotrichum gloeosporioides (accession number AFD31817.1), Mgv1 from Fusarium graminearum (accession number AAM13670.1), Mpk1 from F. oxysporum (accession number RKL49942.1), Smk3 from Sclerotinia sclerotiorum (accession number AOA52648.1), Pmk1 from M. oryzae (accession number XP_003712175.1), and Osm1 from M. oryzae (accession number XP_003714838.1). The sequences were aligned with Clustal Omega and viewed with MView. The red and cyan triangles indicate critical residues of Mps1 for forming hydrogen bonds and hydrophobic interactions with compound A378-0, respectively. The red rectangle indicates the hinge region, which is the boundary of the N-lobe and the C-lobe of these fungal MAP kinases. Download FIG S6, TIF file, 3.4 MB. Copyright © 2023 Kong et al. 2023 Kong et al. https://creativecommons.org/licenses/by/4.0/ This content is distributed under the terms of the Creative Commons Attribution 4.0 International license.

## DISCUSSION

According to the Fungicide Resistance Action Committee (FRAC), there are seven fungicides that target kinases, which can be divided into two groups (see Fig. S7 in the supplemental material). The first group is phenylpyrrole fungicides, including fenpiclonil and fludioxonil, both of which target HOG1 (high-osmolarity glycerol 1) (encoded by the *os-2* gene), a MAP kinase that functions as a central signaling mediator during osmoregulation and responses to external stresses or stimuli of eukaryotic organisms (30–32). The second group is dicarboximides, encompassing chlozolinate, dimethachlone, iprodione, procymidone, and vinclozolin, all of which target Daf1 (decay-accelerating factor 1), a MAP kinase that influences size control, pheromone arrest, as well as the cell cycle (33, 34). However, no structural information is available on the interaction between HOG1 or Daf1 and these fungicides, making the interpretation of their interactions elusive. Actually, to our knowledge, only one complex structure of a fungal kinase and an inhibitor is available in the Protein Data Bank, the structure under accession number 3F3Z. This is a complex of the calcium-dependent protein kinase CDG7 of Cryptosporidium parvum and its inhibitor indirubin_E804 (DRK). DRK also binds to the catalytic pocket of the kinase, similar to A378-0 (Fig. S8A). Close-up views of the superimposed structures show that DRK occupies the left part of A378-0 but is in almost the same position as AMP-PNP, suggesting that the mode of binding of DRK to kinases is similar to that of ATP (Fig. S8B and C).

10.1128/mbio.02883-22.7 FIG S7 Chemical structures of fungicides that target kinases. (A) Fungicides that target HOG1. (B) Fungicides that target Daf1. Download FIG S7, TIF file, 0.5 MB. Copyright © 2023 Kong et al. 2023 Kong et al. https://creativecommons.org/licenses/by/4.0/ This content is distributed under the terms of the Creative Commons Attribution 4.0 International license.

10.1128/mbio.02883-22.8 FIG S8 Comparison of the CGD7/DRK, Mps1/A378-0, and ERK2/AMP-PNP complex structures. (A) Structural superimposition of these structures. The proteins and small-molecule compounds are shown as ribbons and sticks, respectively. (B) Close-up view of A378-0 and DRK from panel A. (C) Close-up view of AMP-PNP and DRK from panel A. In panels A and C, the magnesium ion in the ERK2/AMP-PNP complex that is essential for enzyme activity is shown as a lime sphere. Download FIG S8, TIF file, 2.4 MB. Copyright © 2023 Kong et al. 2023 Kong et al. https://creativecommons.org/licenses/by/4.0/ This content is distributed under the terms of the Creative Commons Attribution 4.0 International license.

The Mps1/A378-0 complex structure that we present in this study clearly elucidated the interactions between the two molecules, making the optimization of A378-0 more readily. In the structure, Mps1 adopts the αC-helix-in and DFG-aspartate-in conformations, both of which are necessary for a kinase to be in the active state (Fig. 3A) (10). In active-form kinases, due to conformational changes during activation, a catalytic spine (C-spine) and a regulatory spine (R-spine) will be formed by residues that are not aligned in the inactive conformation (28, 29). In the Mps1/A378-0 complex structure, residues of the C-spine (residues V37, A50, L107, L155 to L157, I217, and L222) and the R-spine (residues L75, C86, H147, L170, and D210) of Mps1 are aligned and form two spine-shaped structures, supporting an active conformation (Fig. S9). However, with a compact conformation, A378-0 occupies the cleft between the N- and C-lobes of Mps1, which prohibits further conformational changes of Mps1 required for performing its biochemical function (Fig. 2A and C). Therefore, A378-0 could function as a potent inhibitor of the activity of Mps1. Orthologs of Mps1 in other plant-pathogenic fungi are conserved, suggesting the possibility of A378-0 as a hit candidate for the development of a broad-spectrum fungicide (Fig. S6).

10.1128/mbio.02883-22.9 FIG S9 Mps1 is in its active conformation in the Mps1/A378-0 complex structure. (A) Mps1 in the “αC-helix-in” and “DFG-aspartate-in” conformations. Both the side chains of residue E70 of the αC-helix and D167 of the DFG motif (green) point to the catalytic region. (B) The C-spine and R-spine of Mps1 have formed. Residues constituting the C-spine and R-spine are shown as spheres and are in green and gray, respectively. Download FIG S9, TIF file, 2.8 MB. Copyright © 2023 Kong et al. 2023 Kong et al. https://creativecommons.org/licenses/by/4.0/ This content is distributed under the terms of the Creative Commons Attribution 4.0 International license.

It is worthy of consideration that A378-0 might also function on other MAP kinases of M. oryzae. Pmk1 and Osm1 are the other MAP kinases of M. oryzae and share sequence identities of 51.4% and 39.8%, respectively, with Mps1 (Fig. S6). Sequence alignment showed that the critical residues of Mps1 that contribute to A378-0 binding are not all strictly conserved in Pmk1 and Osm1 (Fig. S6). In addition, we were able to obtain a recombinant protein of Pmk1, and we performed SPR assays. The assay results showed nonspecific binding between A378-0 and Pmk1, and thus, no binding affinity could be calculated with any model, suggesting that Pmk1 may not be a target of A378-0 (Fig. S10). Together with the fact that A378-0 does not inhibit appressorium formation, the period during which Pmk1 performs its function, whereas Mps1 does not, it can be concluded that Mps1 is the specific target of A378-0 (Fig. 4A and B).

10.1128/mbio.02883-22.10 FIG S10 SPR sensograms from the assay evaluating binding between A378-0 and Pmk1. The series of concentrations of A378-0 are shown at the right of the charts. Nonspecific binding was observed during the association period, rendering the sensograms unable to be fitted to any model. Download FIG S10, TIF file, 0.2 MB. Copyright © 2023 Kong et al. 2023 Kong et al. https://creativecommons.org/licenses/by/4.0/ This content is distributed under the terms of the Creative Commons Attribution 4.0 International license.

As a compound of the DNA-encoded compound library, A378-0 has been newly synthesized from small compound blocks. Compared with the chemical structures of the ~90 small-molecule protein kinase inhibitors approved by the FDA and the EMA, etc. (see https://www.ppu.mrc.ac.uk/list-clinically-approved-kinase-inhibitors), we can see that the scaffold of A378-0 is absent from them, suggesting that the scaffold of A378-0 is novel and might serve as the basis for the development of new kinase inhibitors. A limitation of this research is that the chemical structure of A378-0 is more complex than those of the phenylpyrrole or dicarboximide fungicides that target two other MAP kinases, suggesting that substantial optimization is necessary to reduce the cost of the production of A378-0 derivatives (Fig. 1B and Fig. S7).

## MATERIALS AND METHODS

### Protein expression and purification.

The recombinant Mps1 (GenBank accession number XP_003712437.1) protein was expressed using Escherichia coli BL21(DE3). The E. coli strain was cultured in lysogeny broth (LB) medium supplemented with 100 μg mL^−1^ ampicillin. The coding sequence of Mps1 with an N-terminal 6×His tag was cloned into the pHAT2 vector (35). Expression was induced with 0.1 mM isopropyl-β-d-thiogalactopyranoside (IPTG) at an optical density at 600 nm (OD_600_) of 0.6, and the cells were cultured at 16°C for 20 h. Cells were harvested by centrifugation and resuspended in lysis buffer containing 20 mM HEPES (pH 7.3) and 400 mM NaCl. After sonication and centrifugation, recombinant protein in the supernatant was collected with nickel-charged resin and further purified with a Superdex 200 size exclusion chromatographic column (Cytiva) equilibrated with storage buffer containing 20 mM HEPES (pH 7.3), 150 mM NaCl, and 2 mM dithiothreitol (DTT). All of the purification procedures were performed at 4°C or on ice.

### Screening for interaction compounds.

Screening was performed according to the manufacturer’s suggestions, with subtle modifications. Briefly, nickel-charged resin was equilibrated with wash buffer (1× phosphate-buffered saline [PBS], 0.05% Tween 20). Twelve micrograms of Mps1 protein was captured with 40 μL of resin and divided in half: one half was used for the screening of the DNA-encoded compound library, and the other half was used as a negative control. The two samples were washed with selection buffer (1× PBS, 0.05% Tween 20, 0.1 mg mL^−1^ sheared salmon sperm DNA) three times and then heated at 95°C for 10 min to release compounds from Mps1 for the next round of screening. The above-described steps were repeated three times, and the compound solution obtained in the last round was used for PCR, sequencing, and analysis.

### Affinity assay.

*In vitro* interactions between Mps1, etc., and A378-0 were analyzed by surface plasmon resonance (SPR). The experiment was performed with the Biacore 8k+ instrument (Cytiva) at 25°C. Compound A378-0 was dissolved in dimethyl sulfoxide (DMSO) at a concentration of 10 mg mL^−1^ (17.7 mM). The running buffer for protein immobilization contained 1× PBS and 0.005% Tween 20, and that for compound analysis contained 1× PBS, 0.005% Tween 20, and 5% DMSO. The flow rates were 10 μL/min for protein immobilization and 30 μL/min for compound analysis. Proteins were immobilized on flow cell 2 of different channels of a series S CM5 sensor chip via an amine-coupling method to 5,000 to 6,000 response units (RU). Flow cell 1 of the corresponding channels of the sensor chip was left blank as a control. Compound A378-0 was injected with a concentration series of 6.25 μM, 12.5 μM, 25 μM, 50 μM, 100 μM, and 200 μM, with one intermediate concentration injected as a duplicate. Both the association and dissociation periods were set to 60 s, and no regeneration was needed. Data were analyzed with Biacore Insight Evaluation software, and values from the first 5 s after the start of the injection were used to calculate the dissociation equilibrium constant (*K_D_*) by fitting to a 1:1 steady-state affinity model.

### Enzyme activity assay.

The kinase activity of Mps1 and Mpk1 was determined with the ADP-Glo kinase assay kit (catalog number V6930; Promega). A peptide (sequence, EGRYRHLVALAT) of Swi6-1 was used as the substrate. The reaction buffer contained 40 mM Tris-HCl (pH 7.5), 20 mM MgCl_2_, and 0.1 mg mL^−1^ bovine serum albumin (BSA). Each sample contained 0.2 mg mL^−1^ protein, 0.1 mM ATP, 0.4 mM Swi6-1, and 0 to 25 μM compound A378-0, with a total volume of 50 μL and a final DMSO concentration of 2%.

The samples were incubated in a 96-well plate at 26°C for 20 min. After that, an equal volume of ADP-Glo reagent was added, and the samples were mixed gently and incubated for 40 min for reaction termination and ATP depletion (no conversion to ADP). Later, a kinase detection reagent was added, and the samples were mixed gently and incubated for another 40 min to convert ADP into ATP. The newly synthesized ATP can be detected using a luciferase reaction, and being measured with a microplate reader (SpectraMax i3x; Molecular Devices) with absorbance at 620 nm. Each sample contained at least three replicates. The amount of newly synthesized ADP indicates the kinase activity of the tested protein.

### Crystallization, data collection, and structure determination.

Mps1 protein in storage buffer was concentrated to 8.5 to 9.5 mg mL^−1^ and then mixed with A378-0 at a molar ratio of 1:1. The protein-compound mixture was incubated on ice for 1 h and then screened for crystals. Crystallization was performed using the sitting-drop diffusion method with the Oryx4 instrument (Douglas) at 18°C. Reservoir buffer was prepared with optimization of the apo-Mps1 crystallizing conditions (PDB accession number 5Z33). The total volume of each drop was 0.5 μL, and the volume ratio of protein to reservoir buffer was 3:7, 5:5, or 7:3. Crystals for data collection were obtained in a reservoir solution containing 20% to 30% Tacsimate, 0.05 M sodium cacodylate trihydrate (pH 6.5 to 7.5), and 0.1 mM spermine. For the collection of X-ray diffraction data, crystals were flash-frozen in liquid nitrogen and protected with reservoir solution supplemented with 20% glycerol.

Diffraction data were collected at Shanghai Synchrotron Radiation Facility Beamline 19U1 (SSRF BL19U1). Data were indexed, integrated, and scaled with HKL-2000 (36). The phase was obtained by molecular replacement with the apo-Mps1 structure (PDB accession number 5Z33) using Phaser MR of the CCP4 software suite (37) and then optimized by iterative refinement with the PHENIX software package (38) and COOT (39). Data processing and refinement results are listed in Table 1. All structural figures were made with PyMOL (40).

### Bioactivity assay.

To evaluate the bioactivity of A378-0 on the pathogenicity and virulence of M. oryzae, we carried out appressorium development, appressorium penetration, and infection assays. M. oryzae strain P131, barley cultivar E9, and rice cultivar Nipponbare were used in these experiments. P131 was maintained on oatmeal tomato agar (OTA) plates (41). The conidial suspension was freshly prepared in a 0.025% Tween 20 solution and diluted as required (42). Compound A378-0 was dissolved in DMSO at a final concentration of 20 mg mL^−1^.

For the appressorium development assay, 20-mm by 20-mm borosilicate glass coverslips were placed into a 9-cm petri dish, and 5 μL of the conidial suspension (2 × 10^5^ to 3 × 10^5^ conidia mL^−1^) with or without A378-0 was then placed onto the glass coverslips. Dishes were incubated in a dark and humid chamber at 28°C for 12 h. Appressorium formation was observed and appressoria were counted with a Nikon microscope.

For the appressorium penetration assay, the back sides of 1-week-old leaves of barley were spot inoculated with 5 μL of the conidial suspension (2 × 10^5^ to 3 × 10^5^ conidia mL^−1^) with or without A378-0 and incubated in a dark and humid chamber at 28°C for 36 h. Appressorium penetration was observed with a Nikon microscope.

For the infection assay, 8-day-old leaves of barley or 3-week-old leaves of rice were used. Before inoculation, the rice leaves were wounded mildly with a needle, while the leaves of barley needed no such pretreatment. The leaves were spot inoculated with 5 μL of the conidial suspension (3 × 10^4^ to 5 × 10^4^ conidia mL^−1^) with or without A378-0 at different concentrations, incubated in a dark and humid chamber at 28°C for 36 h, transferred to an illuminated and humid chamber at 28°C, and incubated for another 3 to 4 days. Lesions were observed and recorded.

### Data availability.

The accession number for the Mps1/A378-0 complex structure in the PDB is 8H59.
