# Supplementary figures and images for: Structure-Aided Identification of an Inhibitor Targets Mps1 for the Management of Plant-Pathogenic Fungi
Source: mBio. 2023 Feb 13;14(2):e02883-22. doi: 10.1128/mbio.02883-22 (PMC10127588; doi:10.1128/mbio.02883-22)

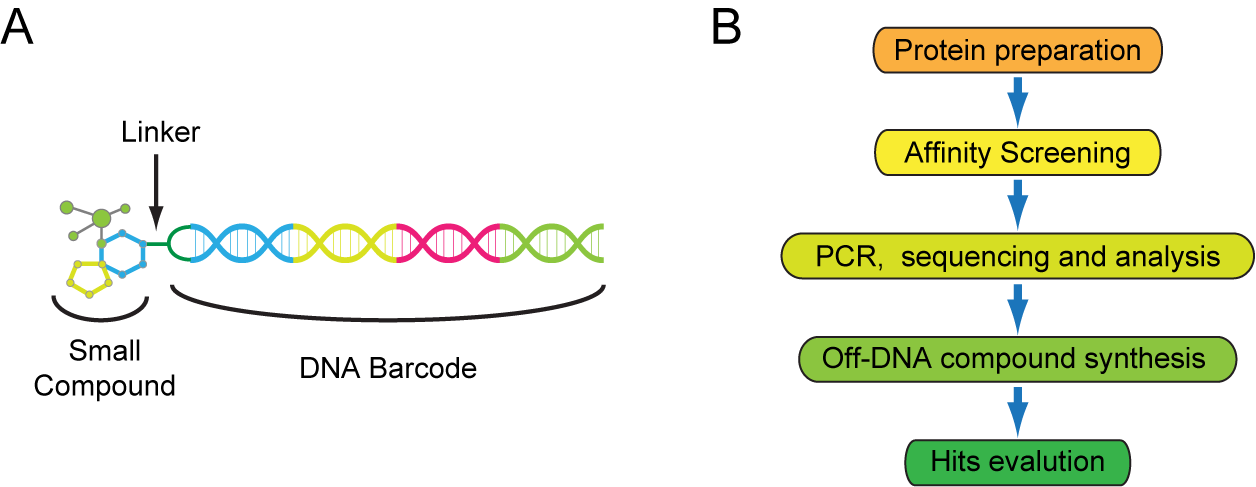

Supplement: FIG S1 [file mbio.02883-22-s0001.tif]

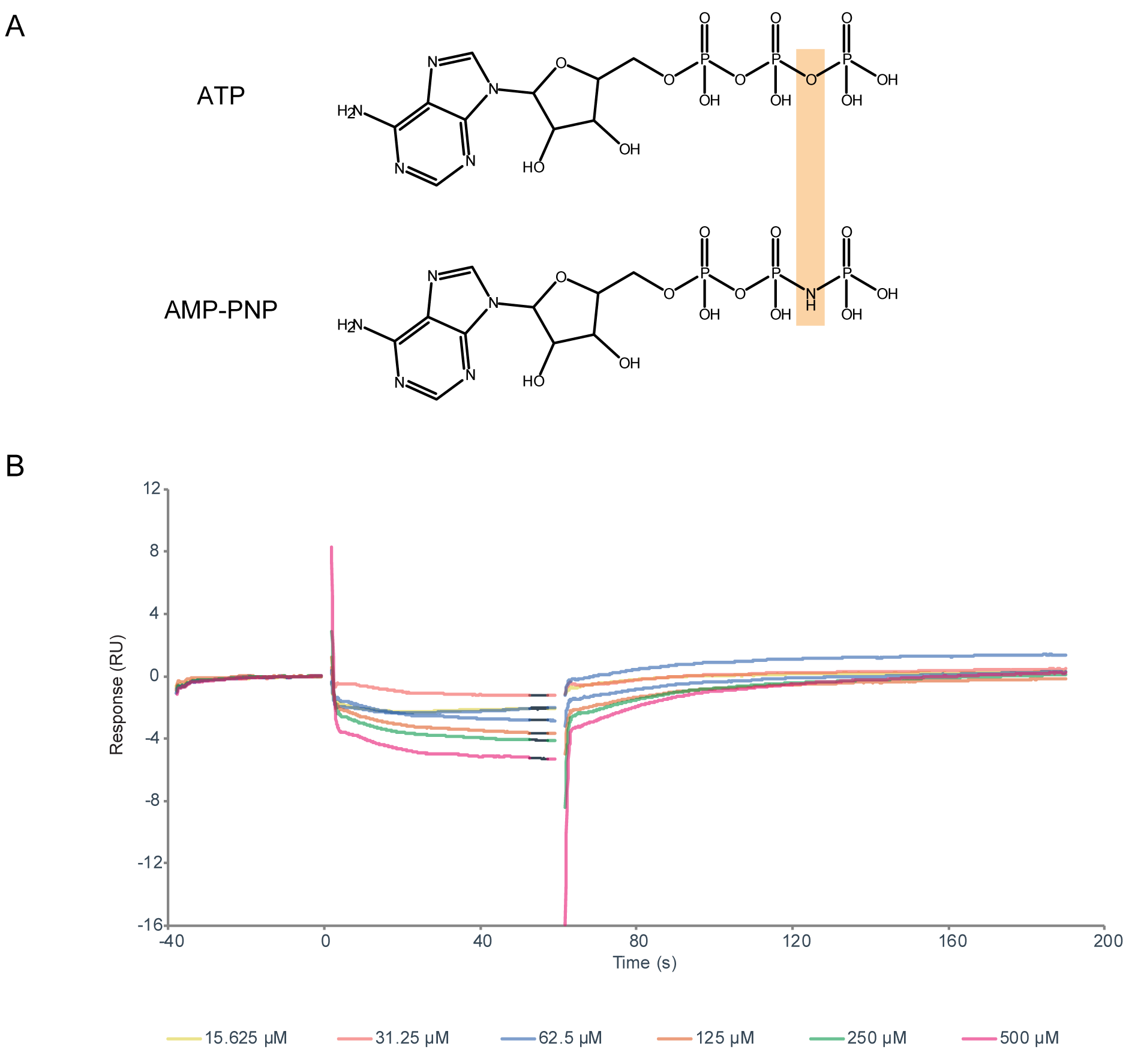

Supplement: FIG S2 [file mbio.02883-22-s0002.tif]

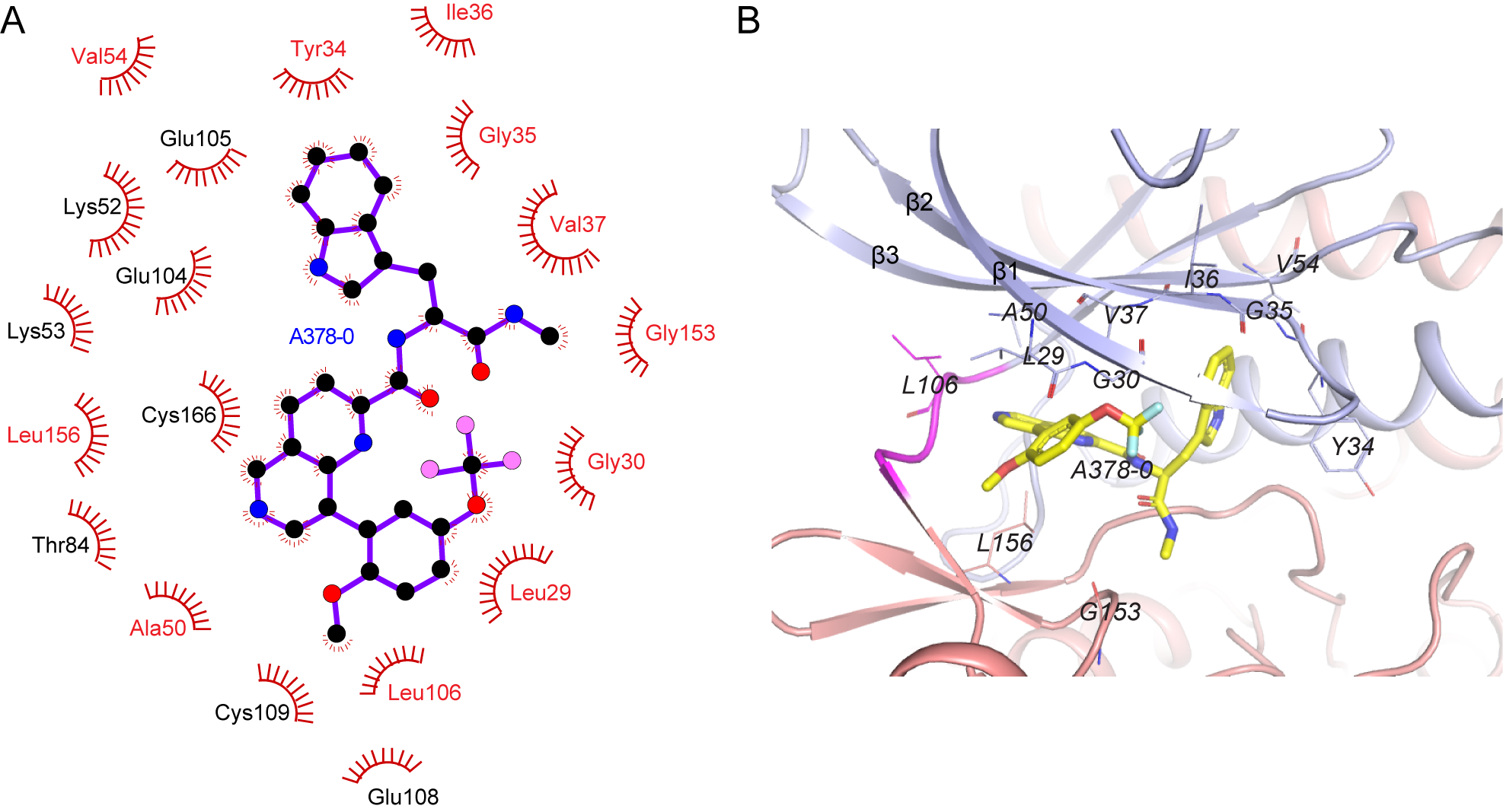

Supplement: FIG S3 [file mbio.02883-22-s0003.tif]

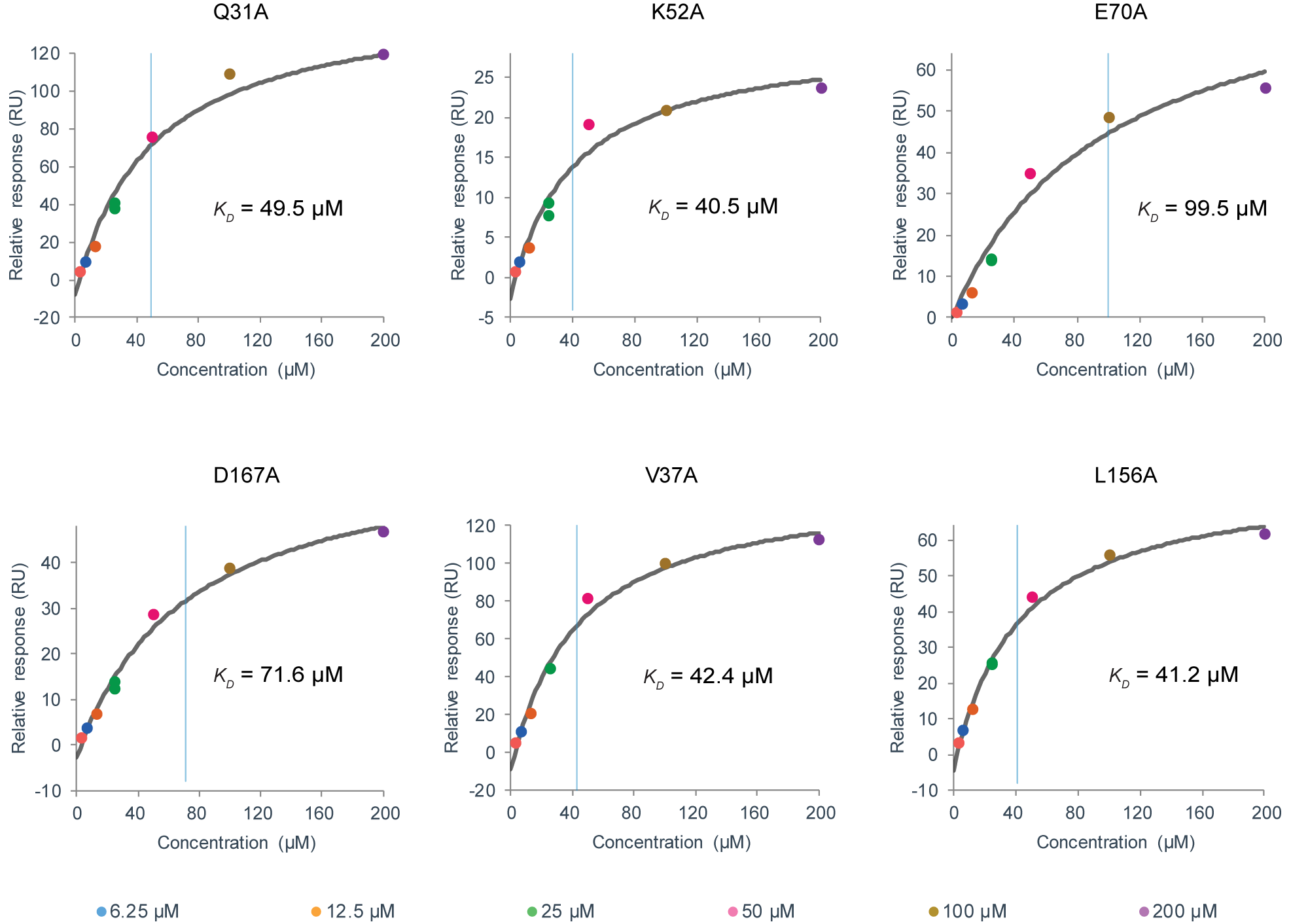

Supplement: FIG S4 [file mbio.02883-22-s0004.tif]

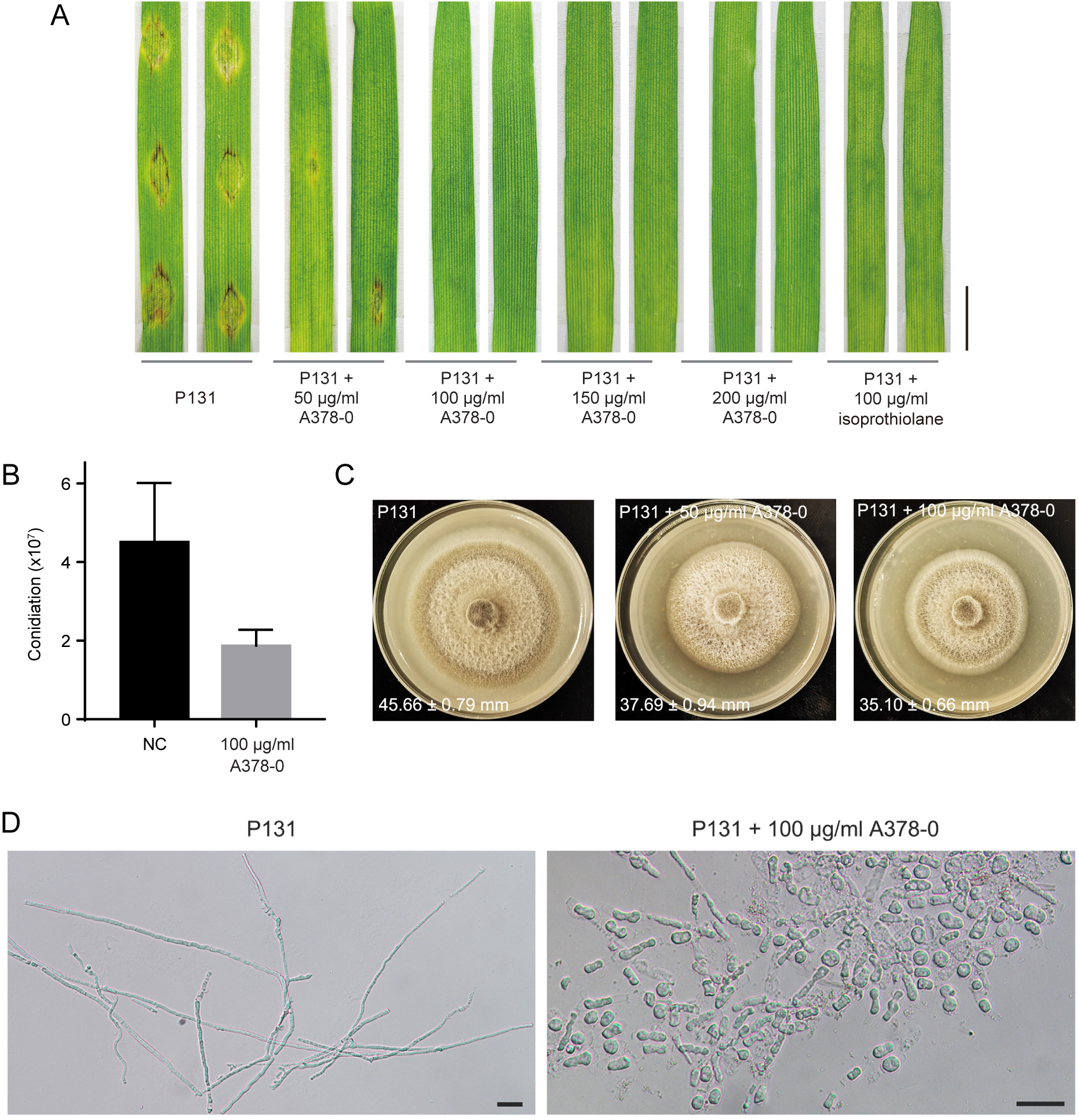

Supplement: FIG S5 [file mbio.02883-22-s0005.tif]

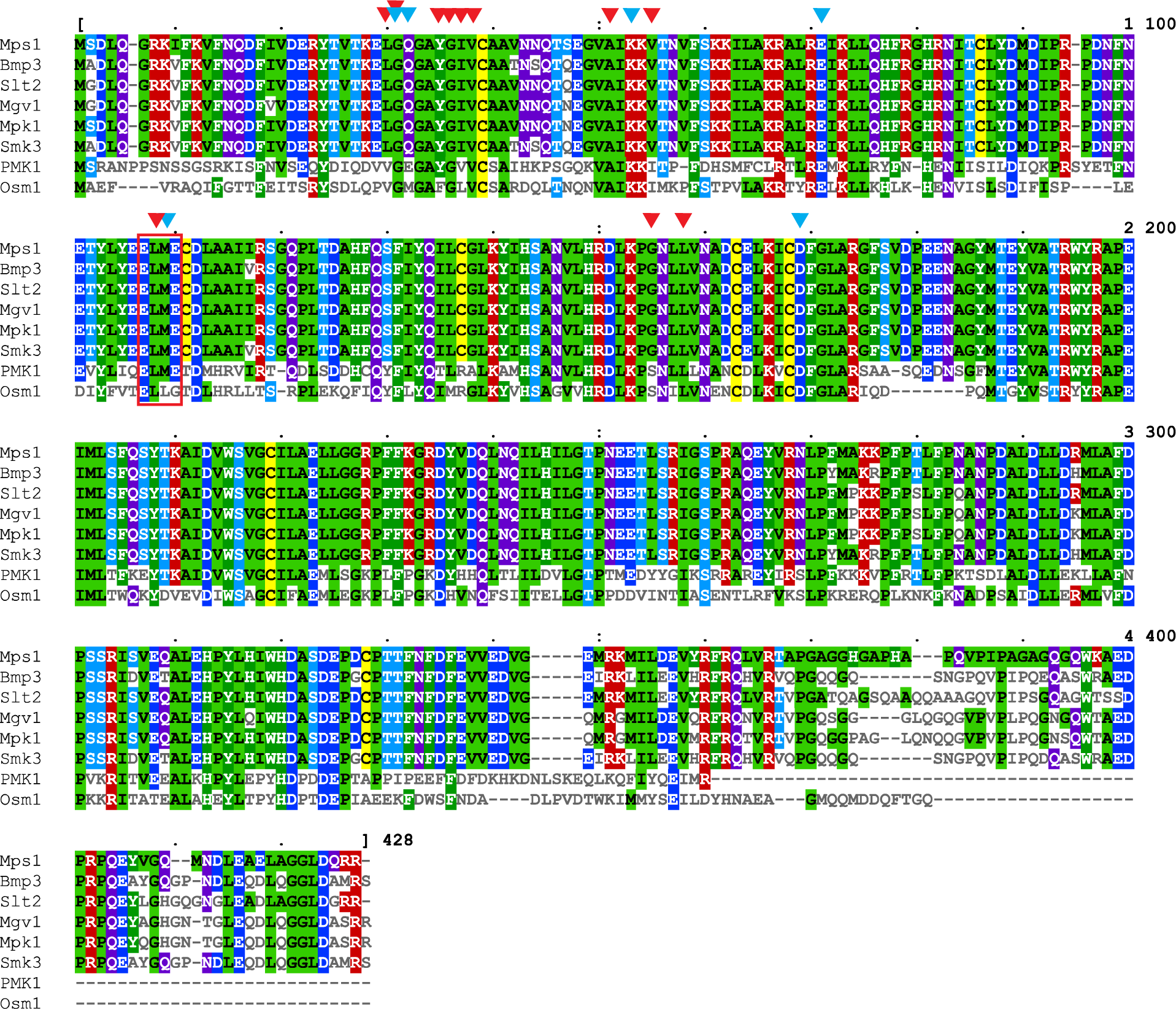

Supplement: FIG S6 [file mbio.02883-22-s0006.tif]

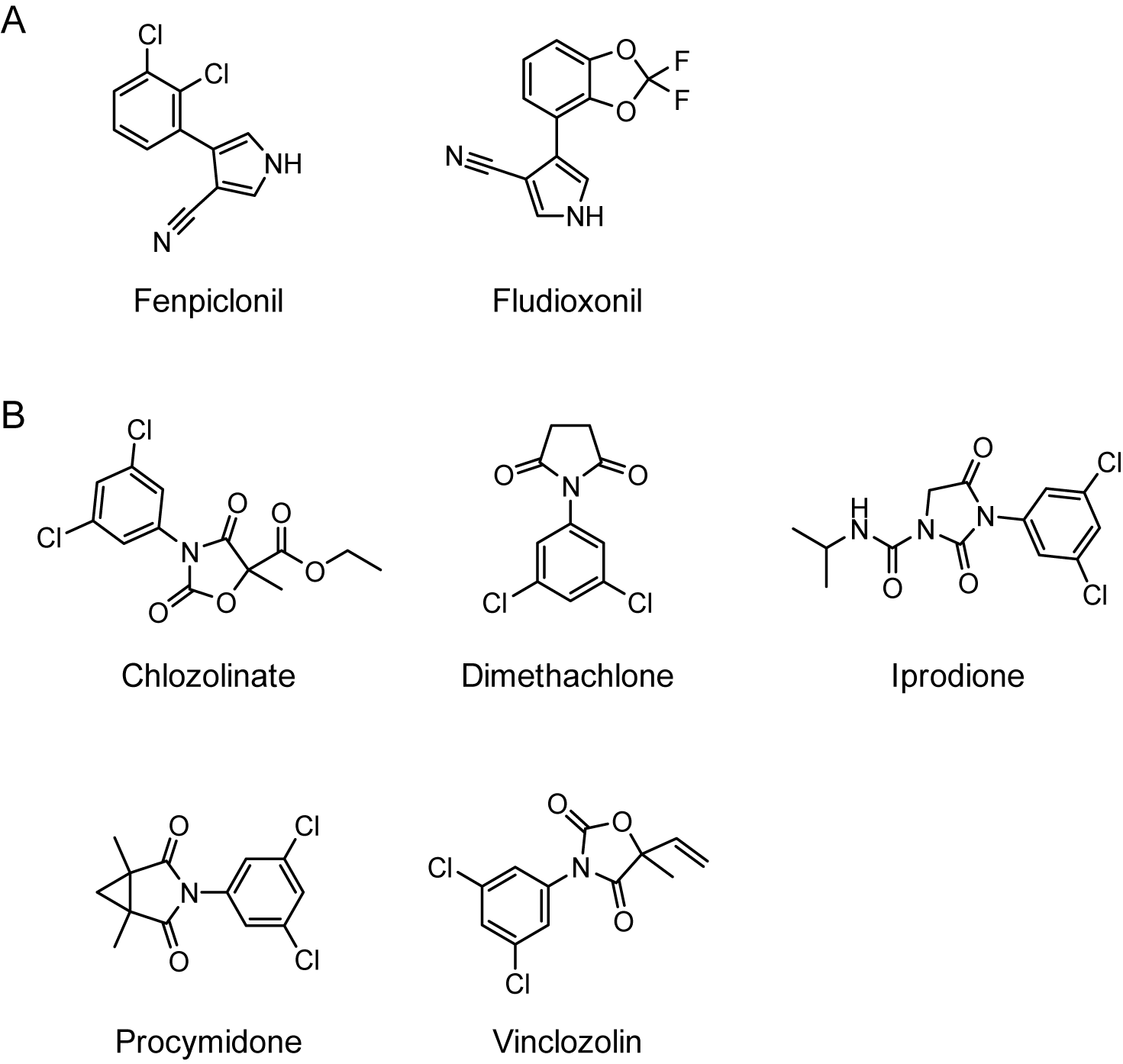

Supplement: FIG S7 [file mbio.02883-22-s0007.tif]

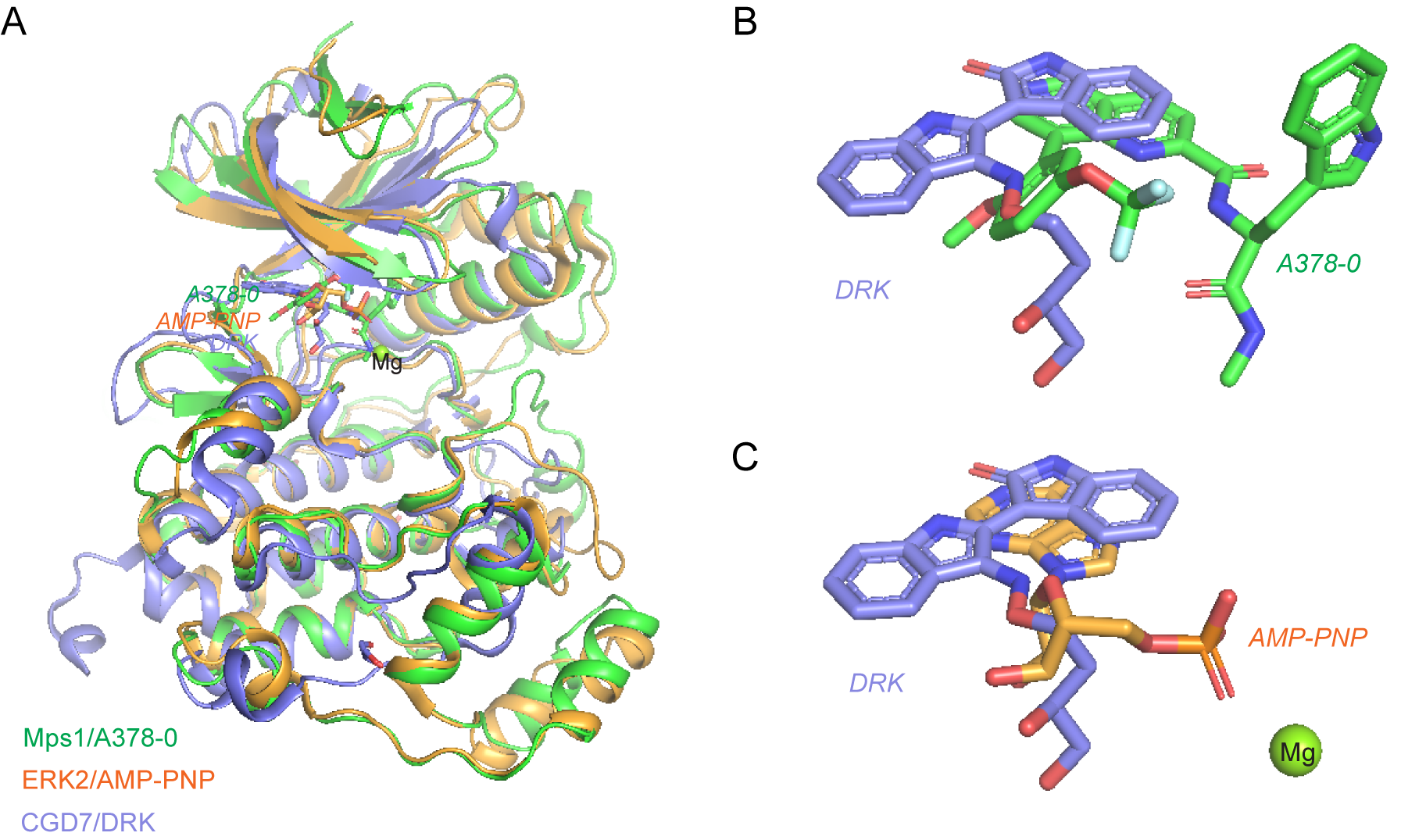

Supplement: FIG S8 [file mbio.02883-22-s0008.tif]

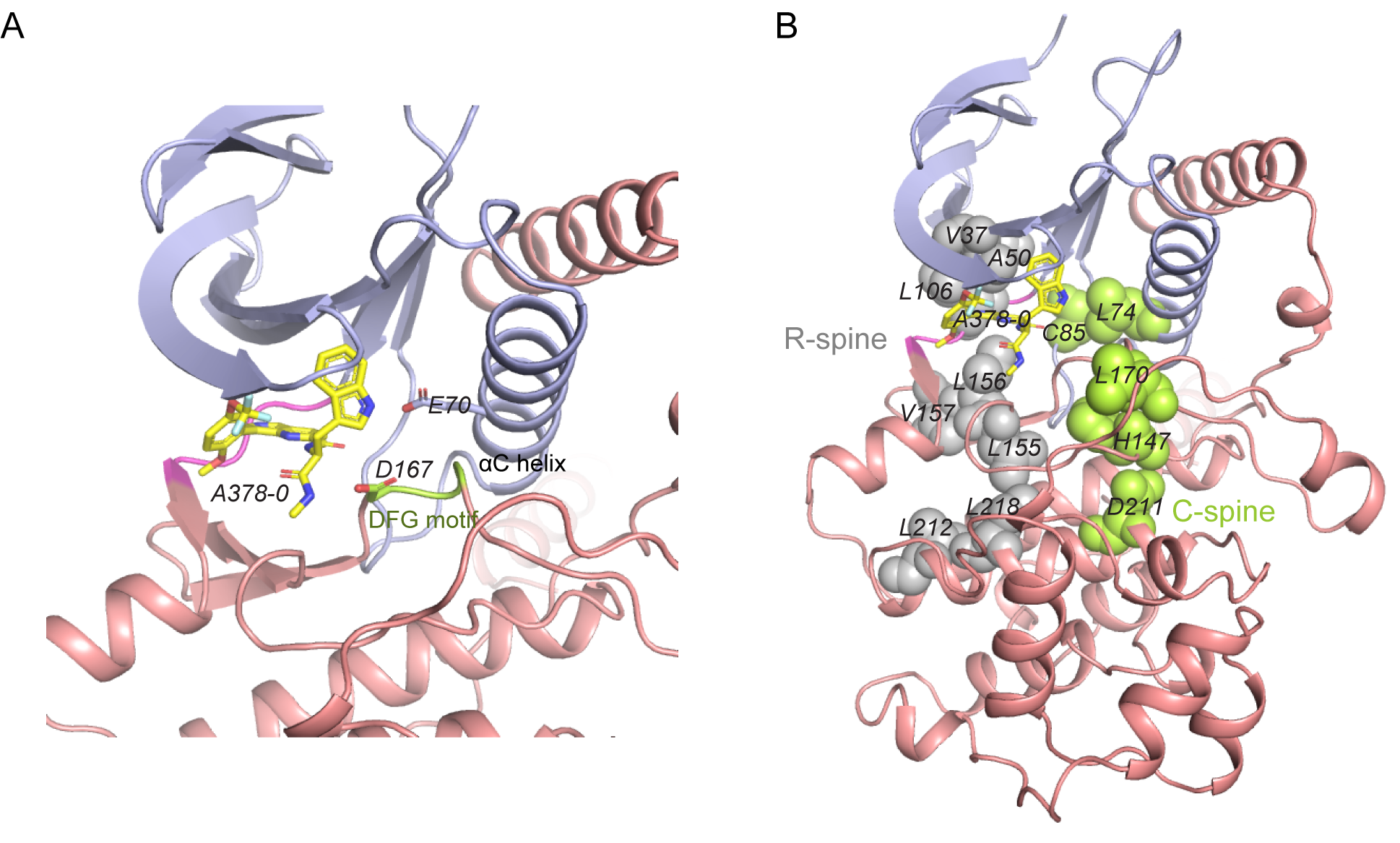

Supplement: FIG S9 [file mbio.02883-22-s0009.tif]

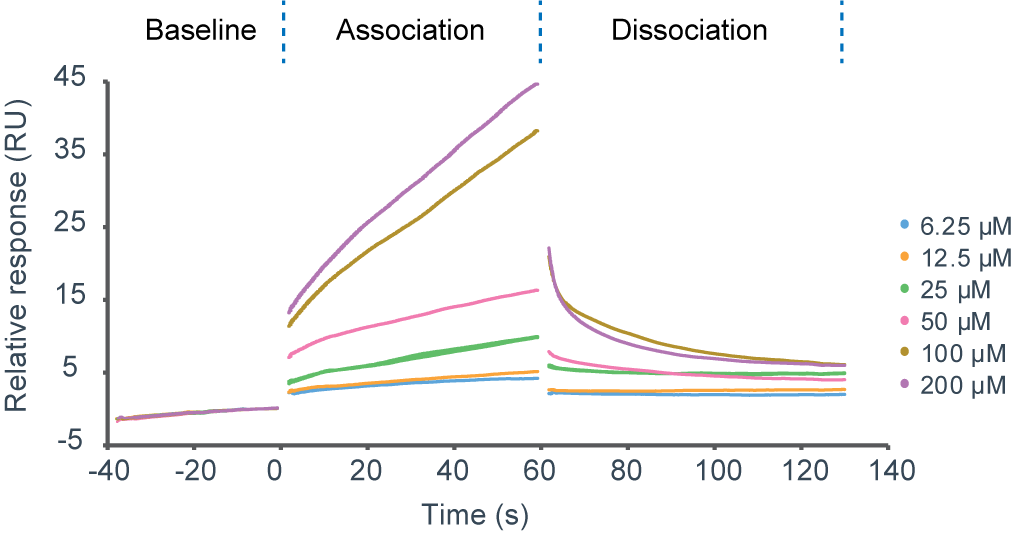

Supplement: FIG S10 [file mbio.02883-22-s0010.tif]
